# Underwater Robot Task Planning Using Multi-Objective Meta-Heuristics

**DOI:** 10.3390/s17040762

**Published:** 2017-04-04

**Authors:** Itziar Landa-Torres, Diana Manjarres, Sonia Bilbao, Javier Del Ser

**Affiliations:** 1 TECNALIA, 48160 Derio, Bizkaia, Spain; itziar.landa@tecnalia.com (I.L.-T.); diana.manjarres@tecnalia.com (D.M.); sonia.bilbao@tecnalia.com (S.B.); 2 Department of Communications Engineering, University of the Basque Country (UPV/EHU), 48013 Bilbao, Bizkaia, Spain; 3 Basque Center for Applied Mathematics, 48009 Bilbao, Bizkaia, Spain

**Keywords:** scheduling, heuristic, multi-objective optimization, random keys encoding, underwater robots, Harmony Search

## Abstract

Robotics deployed in the underwater medium are subject to stringent operational conditions that impose a high degree of criticality on the allocation of resources and the schedule of operations in mission planning. In this context the so-called cost of a mission must be considered as an additional criterion when designing optimal task schedules within the mission at hand. Such a cost can be conceived as the impact of the mission on the robotic resources themselves, which range from the consumption of battery to other negative effects such as mechanic erosion. This manuscript focuses on this issue by devising three heuristic solvers aimed at efficiently scheduling tasks in robotic swarms, which collaborate together to accomplish a mission, and by presenting experimental results obtained over realistic scenarios in the underwater environment. The heuristic techniques resort to a Random-Keys encoding strategy to represent the allocation of robots to tasks and the relative execution order of such tasks within the schedule of certain robots. The obtained results reveal interesting differences in terms of Pareto optimality and spread between the algorithms considered in the benchmark, which are insightful for the selection of a proper task scheduler in real underwater campaigns.

## 1. Introduction

Scheduling problems can be widely conceived as the family of optimization paradigms focused on allocating jobs or tasks over time subject to mutually affecting constraints such as the restricted availability of resources needed to complete the work or a maximum commit time beyond which all tasks should be completed. Scheduling lies at the very core of production processes and operational logistics of many different fields of knowledge, within which the interest in new algorithmic perspectives capable of efficiently dealing with scheduling problems of high dimensionality has become specially notable during the last few years, e.g., manufacturing and service industry [1,2,3], robotics [4], transportation and distribution [5], information processing and communications [6], among others. Within the techniques and tools proposed to cope with scheduling problems meta-heuristics have been extensively exploited as efficient solvers to discover feasible, near-optimal solutions within shorter computation time than exact and/or enumerative methods [7]. Meta-heuristic optimization algorithms have indeed succeeded as efficient algorithmic means to infer near-optimal solutions to complex problems, with a particular emphasis on bio-inspired schemes that leverage and emulate self-learning behaviors observed in Nature when exploring solution spaces. As such, over the last few years studies using Simulated Annealing (SA), Particle Swarm Optimization (PSO), Ant Colony Optimization (ACO) and Genetic (GA) algorithms have been proposed in the literature to solve flow-shop [8] and job-shop scheduling problems [9], with a clear dominance of genetically inspired methods.

In this regard the variety and complexity of scheduling problems has been tackled by the research community from very diverse standpoints. Several works consider static formulation of scheduling problems by which activities are assumed to be known a priori, and constraints are set fixed along time. However, such assumptions rarely hold in practice, where every scheduling problem is likely to undergo unexpected eventualities. This is particularly incident in robotics where, for example, a new activity can be scheduled while the robot is active, or a robot malfunction can be registered due to sensor failures. In such circumstances a new solution must be found—in a preferably small time gap—taking these unexpected events into account and similar to (*incrementally with respect to*) the current schedule. In this application domain the main sources of uncertainty encountered in a real setup can be enumerated as: (1) robot or underwater vehicle malfunctions, including uncertain repair times; (2) increased priority of tasks; and (3) change in due dates, plan, and/or order cancellations, among others. Whenever any of such unexpected event occurs, a new scheduling decision must be made on the reordering of tasks (or new plan with different tasks) in real time. This process is often referred to as “rescheduling”, whose main objective is “to find immediate solutions to problems resulting from disturbances in the system” [10].

In general scheduling problems in real environments do not span a finite set of jobs (static scheduling), but are rather subject to uncertainty and variability that increase further the problem’s computational complexity. In order to face these uncertainties and trace scheduling optimization, dynamic scheduling approaches have been extensively employed in the last years. In this context, authors in [1] presented a learning-based methodology based on machine learning algorithms for dynamic scheduling. In this work the scheduling procedure is split into series of ordered scheduling points. An evolutionary solver provided with dispatching rules was found to outperform at each of such scheduling points, given a state—a set of plant conditions—for the overall system. Since this work plenty of activity has been noted around both static and dynamic scheduling problems, for which machine learning algorithms and other metaheuristics have been utilized [3]. In regards to dynamic scheduling it is also worth to mention the work in [11], where a different approach based on machine learning models for classification is presented. In this case an initial knowledge base was evolved by means of an Evolutionary Algorithm (EA), using results taken from the simulation of the overall production line. By proceeding in this way the scheduling system was able to learn and react against certain unexpected events. Then, a hybrid system composed by neural networks, EA’s and an inductive learner coined as Trace-Driven Knowledge Acquisition (TDKA) was used to extract knowledge about the scheduling process. In this same line of research the authors in [12] developed a hybrid scheduling framework which again consisted of an inductive learning model for releasing jobs within the plant, followed by an evolutionary optimization algorithm for jobs dispatching at the machines. A genetic-based machine learning method and an EA-based status selection scheme have also been employed in [3] to infer optimal scheduling patterns from manufacturing plants.

At this point it is interesting to point out that a scheduling problem can be seen as an optimal selection problem if we consider that the process consists of choosing a subset of tasks or activities from a whole list of possible tasks. Likewise, a scheduling problem can also be formulated by assuming that there is more than one objective—possibly conflicting with each other—to be optimized, yielding a multi-objective scheduling problem. Examples of the possible conflicting arising between different objectives in a robotic environment abound, e.g., battery life versus commit time. In multi-objective optimization problems there exists no single solution simultaneously optimizing each objective function, hence the optimization goal is to efficiently find a set of Pareto-optimal solutions such that any slight improvement in one of the objectives involves a penalty in at least one of the rest of objectives. Generating the Pareto optimal set can be computationally expensive and is not often affordable by means of exhaustive exploration due to the aforementioned complexity of the underlying scheduling scenario. For this reason, a number of stochastically-driven solvers grounded on similar bioinspired heuristics as the ones mentioned previously can be found in the literature to address multi-objective scheduling problems: EA’s, Tabu Search [13], SA [14], Harmony Search [15], and Ant Colony Optimization [16]. While they do not guarantee the identification of solution sets that optimally trade among different objectives of the problem at hand, such techniques attempt at finding a good approximation of Pareto-optimal sets.

In this paper the focus is placed on underwater collaborative task scheduling, in which a group of underwater vehicles (AUVs, ROVs) together with other support vehicles (USVs) collaborate with each other to accomplish a set of tasks. In off-shore and maritime missions there are several scenarios such as the monitoring of chemical pollution, the detection/inspection/tracking of plumes or ocean surveying, where a collaboration among underwater vehicles is required in order to accomplish the scanning of a set of areas. In this proposal, the main idea gravitates on the intuition that, given certain areas to be scanned, an algorithm should be able to calculate which the optimal set of vehicles is and which path they should follow to fulfill the mission tasks optimally in terms of time and cost (e.g., battery level), taking into account restrictions such as the distance to the starting point or underwater currents. Examples of tasks involved in the scanning of an area are “move to waypoint”, “follow row”, “measure” or “take samples” (e.g., from the H${}_{2}$S concentration in the area), “acquire stereo vision data” (images and/or video), “switch on/off equipment”, “send communication”, and other duties alike.

In this scenario several approaches from the recent literature have revolved around multi-robot task scheduling problems. Authors in [17] present a real-time fuzzy-based task scheduler and routing system capable of guiding mobile robots from their source points to their destinations with real-time obstacle avoidance. In [18] a multi-robot task scheduling problem at the coalition level is addressed with heuristics. In the same line of research, authors in [19] propose a multi-agent approach for task allocation and scheduling aimed at minimizing the total execution time. Specifically, the collaboration between swarms of robots for accomplishing a mission is relevant for making it faster or less expensive by means of employing more number of robots with cheaper sensors instead of using a high precision robot. Following this rationale, it is necessary to divide the tasks of a mission and assign them to different robots in an optimized way. In order to cope with this optimization problem, this paper proposes and evaluates multi-objective solvers for optimal underwater collaborative task scheduling. The mathematical formulation of the problem tackled in this work considers optimality as measured by two conflicting criteria: the minimization of the mission cost (accounting for different cost aspects of the schedule such as its impact on the energy consumption of the robots) and the minimization of its total completion time. To efficiently deal with this problem the article explores the practical performance of three different multi-objective heuristic techniques, all resorting to Random-Keys encoding to numerically represent the assignment of robots to tasks and their scheduled execution along time. A real based scenario deployed in Gran Canarias (Spain) will be utilized to assess in practice the performance of the three heuristic schedulers, under different operational situations: a baseline scenario, a battery-limited scenario and a distance-based scenario. Simulation results will evince the practical applicability of the proposed approach to real underwater scenarios subject to cost and total time minimization criteria, and will also unveil performance gaps between the heuristics considered in the benchmark regarding their Pareto spread and optimality.

The paper is organized as follows: Section 2 establishes the mathematical notation of the paper and formally casts the addressed optimization problem, whereas Section 3 and subsections therein provide details on the considered multi-objective heuristics and the solution encoding utilized to represent the schedules. Next Section 4 describes the simulation setup and the considered scenarios, presents and discusses on the simulation results obtained by using the aforementioned heuristics. Finally, Section 5 ends the manuscript by presenting the conclusions extracted from this work and by outlining future research lines.

## 2. System Model

**Figure 1:**
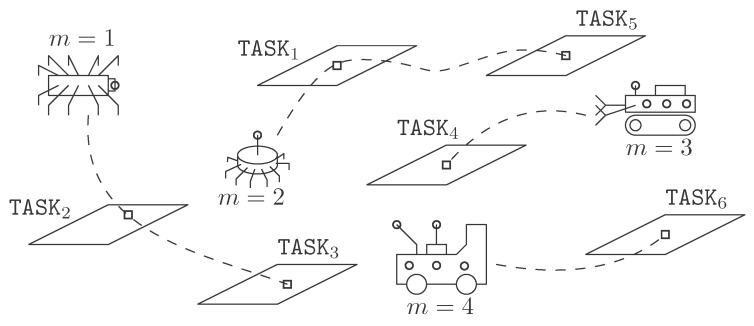
Schematic diagram of the considered scenario for M=4 robots and a mission composed by N=6 tasks.

In reference to Figure 1 we consider an underwater scenario where *M* deployed robotic vehicles cooperate in order to complete a mission composed by *N* tasks ${\left\{{\mathrm{TASK}}_{n}\right\}}_{n=1}^{N}$. Such tasks are assumed to be indivisible (atomic). Each robot may—or not—be qualified to accomplish a certain tasks (due to e.g., the need for special equipment installed on board), for which we define a M×N qualification matrix $Q≜{\left\{{\left\{q_{m,n}\right\}}_{m=1}^{M}\right\}}_{n=1}^{N}$ such that $q_{m,n}=1$ if robot *m* is qualified to accomplish task ${\mathrm{TASK}}_{n}$ (and 0 otherwise). The time required to complete one task varies among robots due to different (yet assumed to be estimable) reasons, such as net speed of the robot itself. This duration will be hence given as $T_{m,n}^{□}$, where the dependence of this time with *n* (task) and *m* (robot) is made explicit.

Differences among robots to complete the tasks come along with a higher operational price (cost) when robots with short completion times are assigned to any task within the mission. This cost collects and represents penalties arising from a more extensive allocation of high-performing robots in favor of shorter completion times of the overall mission. Such penalties can be exemplified by a higher expected energy consumption of the robot when moving quicker through the underwater medium due to the higher dynamic resistance of the water. Cost, furthermore, is also roughly determined by the order in which the task is performed along the scheduling of each robot. The cost incurred by a robot m∈{1,…,M} when performing task $T_{n}$ will be expressed as $0\leq C_{n,m}^{j}<\infty$, where *j* is an integer number denoting the relative position of task ${\mathrm{TASK}}_{n}$ within the task schedule of robot *m*.

With the above definitions in mind, the time at which task ${\mathrm{TASK}}_{n}$ is completed will depend on (1) the robot to which it is assigned; (2) the proficiency under which the allocated robot can perform the task; and (3) the time at which the allocation is effective. The assignment of robot *m* to task ${\mathrm{TASK}}_{n}$ is enforced depending on the schedule designed for the entire operation, which can be defined first by a mapping λ:{1,…,N}↦{1,…,N} of tasks to robots, followed by a second set of mappings $\mu_{m}:\{1,\ldots ,|N_{m}\left|\right\}\mapsto \left\{1,\ldots ,\right|N_{m}\left|\right\}$ (one per robot) that sorts the subset of tasks $N_{m}\subseteq N$ allocated to robot *m* along time (with |·| denoting cardinality). It should be clear that index *j* in the cost term $C_{n,m}^{j}$ is given by $j=\mu_{m}\left(n\right)$. Also it is straightforward to see that $N_{m}≜\left\{\left\{1,\ldots ,N\right\}:\lambda \left(n\right)=m\right\}$.

The time at which robot *m* may start task ${\mathrm{TASK}}_{n}$ (with $n\in N_{m}$), defined as $T_{m,n}^{\Delta }$, must therefore fulfill $T_{m,n}^{\Delta }\geq T_{m,n-1}^{\Delta }+T_{m,n-1}^{□}$, where ${\mathrm{TASK}}_{n-1}$ is implicitly assumed to be the task prior to ${\mathrm{TASK}}_{n}$ within $N_{m}$. Following the same rationale, robot *m* finishes its task commit at time $T_{m,n_{m}^{*}}^{\Delta }+T_{m,n_{m}^{*}}^{□}$, where $n_{m}^{*}$ represents the index of the last item in the subset $N_{m}$ of tasks assigned to robot *m*. By using this notation, the total time taken to complete the mission will be given by

(1) $$T_{mission}≜\max_{m\in \{1,\ldots ,M\}}\left(T_{m,n_{m}^{*}}^{\Delta }+T_{m,n_{m}^{*}}^{□}\right),$$

i.e., the maximum time needed for the pool of robots to complete all compounding tasks of the mission at hand. It is here implicitly assumed that no task is left unassigned. Likewise, the operational cost of the mission will be given by the sum of all costs incurred by the scheduling (λ,μ) when allocating tasks to robots and ordering them along time, i.e.,

(2) $$C_{mission}≜\sum_{m=1}^{M}\sum_{n=1}^{N}q_{m,n}I\left(\lambda \left(n\right)=m\right)C_{n,m}^{\mu_{m}\left(n\right)}=\sum_{m=1}^{M}\sum_{n\in N_{m}}q_{m,n}C_{n,m}^{\mu_{m}\left(n\right)},$$

where I(·) equals 1 if its argument is true and 0 otherwise. The multi-objective problem to be tackled in this manuscript is therefore the simultaneous minimization of $T_{mission}$ and $C_{mission}$, subject to λ being a one-to-one mapping (i.e., tasks can be only assigned to one robot and cannot be parted anyhow) and $T_{m,n}^{\Delta }\geq T_{m,n-1}^{\Delta }+T_{m,n-1}^{□}$ (corr. no task can start before its assigned robot finishes processing the previous work in its schedule).

## 3. Considered Algorithms

In order to efficiently solve the simultaneous minimization of the mission completion time and cost respectively given in Expressions (1) and (2) we will explore the use of several multi-objective meta-heuristics, which are detailed through the following subsections.

### 3.1. Multi-Objective Harmony Search Algorithm (MOHS)

We start by delving into the first solver considered in this study, the Harmony Search (HS) optimization algorithm, which was coined by Geem et al. in [15] and subsequently applied to several applications and problems springing from diverse disciplines, such as Energy [20], Transport [15,21], Games [22] and Health operations [23], among many others [24].

In this paper we focus on deriving a multi-objective version of the HS algorithm that attempts at simultaneously minimizing the aforementioned fitness functions: total time and cost. Due to its population-based search procedure, HS operates on a set of candidate solutions ${\left\{H\left(k\right)\right\}}_{k=1}^{K}$ (denoted as Harmony Memory in related works), which are iteratively modified towards regions of progressively higher optimality by means of combination and mutation operators applied to each of their compounding variables. Assuming the classical notation related to HS, we will hereafter refer to a possible candidate set H(k) as *harmony*, whereas *note* denotes any of its compounding *N* entries. In our optimization framework each note is encoded based on a Random-Keys (RK) strategy [25], by which each note $H_{k,n}$ (with n∈{1,…,N} and k∈{1,…,K}) is represented as a real positive number whose integer part $\left\lfloor H_{k,n}\right\rfloor$ denotes the index of the robot assigned to accomplish task ${\mathrm{TASK}}_{n}$, and whose fractional part $H_{k,n}-\left\lfloor H_{k,n}\right\rfloor$ identifies the relative order of the tasks. Tasks with lower fractional part are therefore executed earlier than those with higher fractional part.

To decode a RK-encoded individual all notes sharing the same value for their integer part are grouped and sorted in increasing order of the value of their fractional part. This process results in the planning of tasks for every robot. For instance, the solution vector $H\left(k\right)≜{\left\{H_{k,n}\right\}}_{n=1}^{4}=\left\{2.35,\;1.96,\;2.73,\;1.14\right\}$ corresponds to the schedule:

$$\mathrm{Robot}1:\;{\mathrm{TASK}}_{4},\;{\mathrm{TASK}}_{2}\;\mathrm{Robot}2:\;{\mathrm{TASK}}_{1},\;{\mathrm{TASK}}_{3}$$

In the literature RK has been utilized to represent solutions of evolutionary solvers that handle task plans, often improved further by means of local search procedures [26] or other hybridized optimizers such as PSO [14]. In [15] a fuzzy reformulation of the problem tackled in this latter work was addressed, which extended prior work by adding availability constraints due to preventive maintenance and breakdowns. In [16] the authors designed a genetically inspired algorithm to discriminate optimal decision rules to be imposed within manufacturing systems. Other contributions have also resorted to RK for job-shop problems where a computer simulation of the plant provides a quantitative measure of the optimality fitness that guides the search process [27]. The actual proposed algorithm is based on a previous RK-HS based approach [28] but incorporates a multi-objective approach for obtaining a wide set of solutions.

#### Improvisation Operators

The improvisation procedure of the multi-objective HS solver is mainly driven by two operators, which are sequentially applied to each note with a certain probability yielding a new set of candidate solutions, namely:

- The *Harmony Memory Considering Rate* (HMCR∈[0,1]), which sets the probability that the new value of a given note is drawn uniformly at random from the values of the note at hand in the rest of harmonies. Besides, with a probability of (1-HMCR), the decision variable values are randomly chosen according to their possible range of values. This case is known as random consideration as it increases the diversity of the solutions so that global optimality can be attained. Note that in our designed HS solver the HMCR operator is only applied to the integer part of the note so that changes only affect to the robot’s assignment to each task. Besides, with probability (1-HMCR) the new value is uniformly selected at random from the discrete set {1,…,M}, where we recall that *M* stands for the total number of robots.
- The *Pitch Adjusting Rate* (PAR∈[0,1]) establishes the probability that the new value for a given note is obtained by slightly perturbing its previous value. The PAR operator is only applied to the fractional part of every note so that it changes the order of the tasks. Specifically, a bandwidth BW∈R[0,1] defined beforehand as an additional control parameter of the algorithm performs the pitch adjustment as
  (3) $$H_{k,n}\leftarrow H_{k,n}+x\cdot BW,\;\;\left(\mathrm{with}\;\mathrm{probability}\;\mathrm{PAR}\right),$$
  where *x* is the realization of a discrete random variable taking values from the alphabet {−1,+1} with equal probability.

Once new harmonies have been improvised, they are evaluated in terms of the two objective functions (mission completion time and cost) for every improvised melody, and the best (with respect to fitness values and spread) *K* harmonies – out of the newly produced ones and those from the previous iteration – compose the Harmony Memory that lay the basis for new improvisations in the next iteration. The procedure is iterated for a fixed number of iterations I. In reference to Algorithm 1, the steps of the proposed multi-objective HS algorithm are described next:

- The *initialization* process is only executed at the first iteration. At this step, the entries of the Harmony Memory H(k) are randomly generated. The integer part, which identifies the robot that executes task *n* with n∈{1,…,N}, is taken uniformly at random from {1,…,M}. On the other hand, the fractional part – which identifies the order of the tasks – is also randomly picked from the range R[0,1).
- In the *improvisation* procedure, the two probabilistic operators described above are sequentially applied to each note so as to produce a new set of *K* improvised harmonies.
- Both the total completion time and the cost as per Expressions (1) and (2) are evaluated for each newly generated candidate solution.
- Based on such metric values, a rank and a crowding distance value are assigned at each solution. As explained in [29] candidate solutions with less rank value and largest crowding distance value are preferred in order to fill the harmony memory for subsequent iterations. That is, between two solutions with different non-domination ranks, the point with the lower rank is selected. Alternatively, if both points belong to the same front the point located in a region with lesser number of solutions (i.e., larger crowding distance) is preferred.
- If the number of iterations is less than I, the algorithm iterates by returning to step B. Otherwise, the algorithm stops and the set of candidate solutions that compose the estimated Pareto front is declared as the proposed solution to the underwater collaborative scheduling problem posed in this manuscript.

**Algorithm 1:** Multi-objective Harmony Search Algorithm (MOHS).
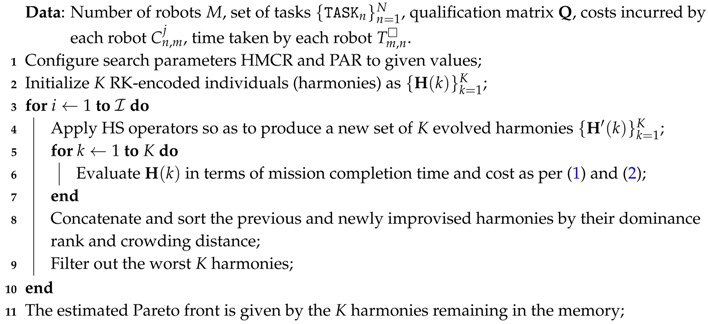


### 3.2. Non-Dominated Sorting Genetic Algorithm-II (NSGA-II)

Originally contributed by Deb et al. in [27], the NSGA-II solver utilizes a non-dominated approach and a crowding distance criterion similar to the ones used in the multi-objective HS scheme detailed above to solve multi-objective optimization problems. At every step of the search process described in Algorithm 2 the NSGA-II algorithm ranks the possible solutions with respect to each of the objectives, organizing them into fronts or sets of non-dominated solutions. The difference with respect to its HS-based counterpart lies on the operators utilized for producing new candidate individuals: in this paper a blend crossover and a Gaussian mutation operator are used to evolve the candidate solutions at each iteration. Elitism is implemented in the selection process, allowing the best found solutions so far (i.e., the Pareto front) to always remain within the surviving pool of candidates. As mentioned in the introduction, individuals will be represented by adopting the RK-based encoding strategy detailed for the HS-based solver.

NSGA-II has been widely utilized in scheduling. For example, in [30] an hybrid multi-objective evolutionary approach based on NSGA-II is proposed in which release times and energy savings in steel plants are optimized. Also related to resource allocation, in [31] emphasis is given to the optimization of two Quality-of-Service (QoS) parameters (makespan and availability of the grid system) for the scheduling of tasks in a grid. Required jobs are assigned to nodes within a computation grid towards optimizing several indicators of the quality of service under which such jobs are produced. The latest advances in this type of problems are related to reconfigurable manufacturing systems (RMS), a concept of active research within the field of manufacturing systems framed in the context of mass customization. A RMS is able to physically and/or logically change its configuration in order to implement the specific functionalities and capacities required by every scheduling period. The main goal is to accomplish a proper scheduling of multiple products to operate a reconfigurable system in a cost-effective manner. The two conflicting objectives are the minimization of the total costs (including capital and reconfiguration investments) and the minimization of the total tardiness [32].

### 3.3. Pareto-Archived Evolution Strategy (PAES)

PAES [33] is a multi-objective evolutionary algorithm shown to obtain remarkable Pareto fronts with a lower computational complexity than other multi-objective heuristics. This solver comprises three steps broken down in Algorithm 3: (1) the generation of a candidate solution; (2) the mutation of such a solution to obtain a new candidate individual; and (3) the replacement of the original solution with the mutated individual if the former is dominated by the latter, or add the mutated individual to the archive of non-dominated solution if it is dominated by no solution contained in the archive. This archive is split into a number of folds or regions of equal size for which a crowding degree value is determined by counting the solutions falling within each region. This approach employs a Gaussian mutation and prioritizes candidate individuals associated to poorly crowded regions so as to provide diversity in the Pareto front. Once a maximum number of iterations is met PAES terminates and the archive includes the set of solutions that form the final estimation of the Pareto front. The PAES algorithm has been applied in scheduling problems such as the job-shop scheduling approach in [34] in which PAES is compared to other multi-objective solvers.

**Algorithm 2:** Non-Dominated Sorting Genetic Algorithm-II (NSGA-II)
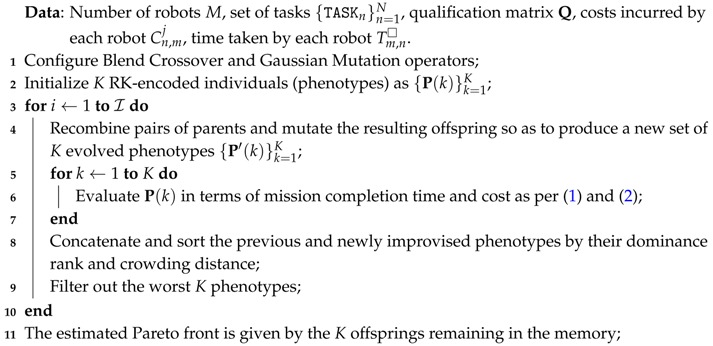


**Algorithm 3:** Pareto-Archived Evolution Strategy (PAES)
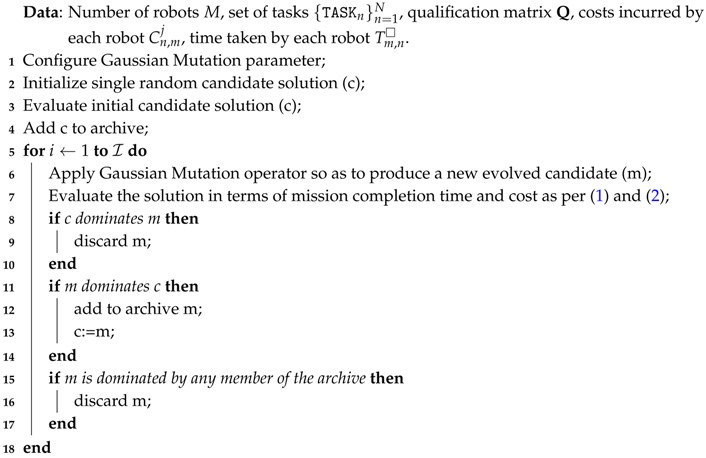


## 4. Simulation Setup and Experimental Results

In order to assess the performance of the above multi-objective solvers when applied to the underwater collaborative scenario described in preceding sections several computer simulations have been carried out over different hypothesis posed over the operational circumstances under which the deployed robots operate. Such simulation scenarios are described next.

### 4.1. Simulation Setup

The aim of the real simulation setup presented in this paper is to handle several robots and plan their actions so as to optimize the whole mission costs.

**Figure 2:**
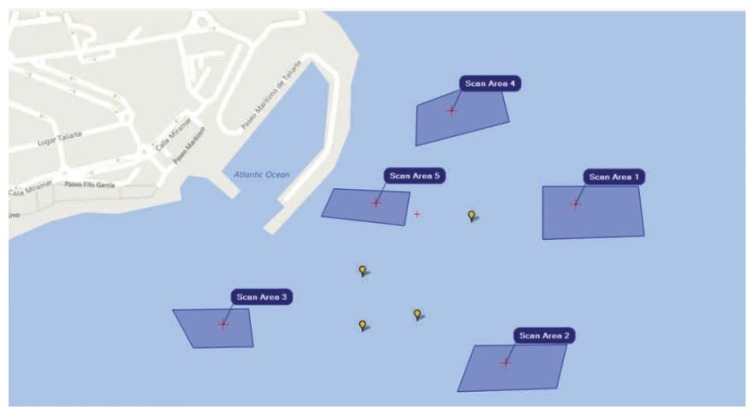
Real simulation setup deployed in Gran Canarias (Spain).

The site used for the trials is the open sea waters of the Melenara Bay located on the East coast of Gran Canaria (Coordinates: Latitude $27^{\circ }$ 59.046’ N: Longitude: $15^{\circ }$ 22.118’ W). Regarding mission planning in underwater operations, it can be studied at two different levels: high-level and low-level. This paper focuses on high-level mission planning which consists of the schedule and breakdown of tasks that need to be performed by a swarm of AUVs in order to accomplish a specific mission. In maritime operations, high-level tasks can be grouped by 3 categories: point, column or area. “Point” tasks start and end at the same location (e.g., take a photo or release small rock at a given location); “column” tasks are executed along a row (e.g., measure H${}_{2}$S at different depths along a water column); “area” tasks are executed while covering an area (e.g., cover area *A* and record video to detect objects). For this demonstrator, the focus is put on “area” tasks related to a “seabed mapping” scenario, in which the mission comprises scanning the area, taking measurements, navigating and providing information to the operator. The exchanged information includes diverse actions such as the acquisition of images and/or videos, the delivery of measurements from different sensors onboard or even additional parameters acquired by equipment that is switched on or off on demand. That being so, to define a seabed mapping scenario, apart from defining the tasks that comprise the mission, the first step is to define the set of available robots and the areas of interest, as shown in Figure 2. The algorithms under consideration make it possible to select a subset of robots to complete the mission, given their associated time and cost estimates. The type of tasks that each robot may acomplish are:

- ${\mathrm{TASK}}_{1}$: “move to waypoint”.
- ${\mathrm{TASK}}_{2}$: “Follow a row”.
- ${\mathrm{TASK}}_{3}$: “Measure or take samples”.
- ${\mathrm{TASK}}_{4}$: “Acquire images and/or video”.
- ${\mathrm{TASK}}_{5}$: “Switch on/off equipment”.
- ${\mathrm{TASK}}_{6}$: “Send information”.

Differences among robots are related not only to their capabilities to perform the taks (i.e., not all robots can accomplish any task), but also to their price and incurred cost when undertaking a certain task; each robot may require different time and battery cost to carry out a certain task. Moreover, it is important to note that by means of dividing the global area into subareas and enabling AUVs cooperation, the whole area could be covered performing a faster real-time mapping and inspection of the area.

**Figure 3:**
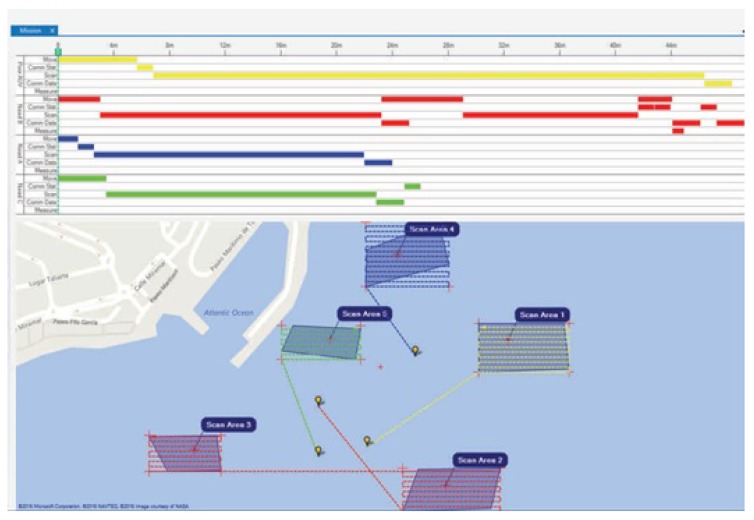
Results obtained with the GUI interface.

As mentioned in Section 3, the algorithms are used to generate the global high-level mission plan in three different situations. The first case is the baseline scenario which simulates the preparation of the mission before it starts. In this situation, there are no time constraints and an optimal solution is desirable. The output plan of the algorithm is given to the AUVs which are deployed over the seabed to be mapped. The second case is a battery-limited scenario. This scenario can occur once the mission is being carried out and a replanning needs to be done due to an unexpected event. In this case, the battery level of the AUVs is limited as they have been underwater for some time. The third case that is being considered is a distance-based scenario. This situation is of interest when the equipment of an AUV is damaged during the mission and the algorithms need to reschedule the plan and decide whether to distribute the tasks between the participant AUVs or include a new AUV depending on its location. Thus, the output provided by every scheduler is the task plan for each robot, which includes their task sequence and planned trajectory. The computed plans are shown in a mission management graphical user interface. For this setup, all those tasks are addressed with a graphical user interface that show the mission input and output on a geographical information system, along with a Gantt chart of the task schedule for each robot, as depicted in Figure 3.

Once the algorithm has optimized the task schedule for each member of the entire robotic swarm, the operator selects the best candidates among the proposed solutions. The output of the solver, now integrated into the human-machine interface, has revealed that they are capable of planning the mission of up to four robots, covering diverse areas to be scanned with computation time in the order of a few minutes’ computation time. In summary, the GUI entails the following steps:

- Mission definition: the operator draws on a map in the GUI the set of areas to be mapped. The system informs the operator which robots are available and their configurations.
- Multi-objective scheduling algorithm: to optimize the whole mission and coordinate the robots, the system needs a planning model describing the available objects and their possible tasks. Given this model, the proposed heuristic algorithms compute the best sequence of actions performed by the robots, i.e., the plan.
- Gantt chart view of the task plans for each robot: the mission plan consists of a list of tasks assigned to each robot. Plans are shown as paths on the map and Gantt charts, showing the duration of the tasks and the order of execution for each robot.

Since this study is focused on the multi-objective scheduling algorithms themselves, the results provided in the next subsection describes quantitatively the task schedules produced by each of the algorithms.

### 4.2. Experimental Results

In order to assess the performance rendered by all multi-objective approaches proposed in this paper, namely MOHS, NSGA-II and PAES, a comparison study in a real scenario in Gran Canarias (Spain) will be presented and discussed. In this real scenario a total of M=4 underwater robots are employed for accomplishing a specific mission composed of different tasks (N=206). As stated in Section 4.1 each robot is capable of executing certain tasks based on its capacity and properties. As robots have distinct functionalities and usages, a different cost and time is associated per pair (m,n), i.e., robots with higher cost per task require less time to execute the task. However, there are tasks that can be performed by different robots, and the selection of one or another depends on the total of list of tasks and the availability of each robot.

Simulation results consider: (1) a baseline scenario in which robots are located relatively close to each other and without battery limitations; (2) a battery-limited scenario in which robot m=4 undergoes a severe battery capacity restriction; and (3) a distance-based scenario in which robot m=3 is located far from the mission area. All multi-objective approaches are configured with the same number of Monte Carlo simulations, i.e., 20 in all cases, and maintain a memory or archive of 50 candidate solutions. This ensures fairness in the comparison between such approaches as the number of fitness evaluations is the same among solvers. The values of the operators for all approaches have been optimized in order to obtain the best performance in the baseline scenario, and are extended to the remaining use cases (battery-limited and distance-based scenarios). Regarding MOHS, the values of the HMCR and PAR operators are set to 0.7 and 0.3, respectively. NSGA-II employs a Gaussian mutation with probability of 0.1. Finally, PAES results are obtained with a Gaussian mutation probability of 0.1.

There are different complementary multi-objective performance metrics that can be employed in order to evaluate the quality of the approximated Pareto fronts obtained by multi-objective approaches. On one hand, cardinality metrics refers to the number of solutions that exists in the resultant Pareto Front; intuitively, a high number of solutions—and hence a high value of such metrics—is preferred. In this context, Table 1 presents the number of non-dominated solutions in the resulting Pareto Fronts per multi-objective approach and use case scenario. As can be shown, MOHS and NSGA-II obtain the highest number of non-dominated solutions, but in distance-based scenarios, when some robots are located far away from each other, only MOHS is able to obtain a wide range of distinct solutions. This is due to the explorative capability of MOHS, which allows exploring solutions in the search space where some robots (the farthest ones) are left out of use. As a result, this solver obtains more diversity of results by means of combinations of different number of robots. Both NSGA-II and PAES utilize the 4 robots in all candidate solutions and as a result, a less number of non-dominated solutions is achieved with both techniques.

In terms of diversity metrics, Table 2 and Table 3 show the normalized hypervolume (HV) metric (%) with a reference point per multi-objective approach in Table 2 and with a common reference point per real case study simulation in Table 3. It is widely known that distribution and spread in multi-objective techniques are a highly sought characteristic: distribution refers to the relative distance among solutions, whereas spread stands for the the range of values covered by the estimated Pareto front. In this regard the HV metric, which calculates the fraction of space covered by solutions in the objective space with respect to a cuboid given by reference points, blends both aspects together into a single numerical score. The results obtained in most of the scenarios reveal a higher HV value when employing the MOHS approach as opposed to its PAES and NSGA-II counterparts. As argued before, MOHS has a better explorative behavior that permits to explore a wider range of solutions with different number of robots, i.e., solutions with similar cost metric values as per Expression (2) but that require slightly more time—corr. (1)—to accomplish the same mission. PAES includes the four robots in all solutions without taking care of the cost metric increment that involves utilizing robots that are far away from the mission area. NSGA-II offers more diversity of solutions than PAES but renders a worse performance than MOHS in terms of the HV metric.

Finally, the coverage rate metric (%) presented in Table 4 reflects the number of solutions within each Pareto Front that are non-dominated by any solution in the rest of fronts. As shown in this table NSGA-II achieves a highest percentage of dominating solutions in its estimated Pareto front. However, when referring to distance-based restricted scenarios MOHS is capable of obtaining the highest percentage of non-dominated solutions due to its capability to explore the search space, by which different number of robots are considered ultimately relaxing the Pareto pressure over the cost metric without penalizing excessively the timing of the mission.

## 5. Conclusions

This work has formulated a joint task assignment and scheduling problem framed within monitoring and inspection underwater missions performed collaboratively by robot swarms. Optimality in this problem is defined by the minimization of two conflicting criteria: the completion time of the mission and its cost, the latter defined as a numerical score of the impact that the assignment and scheduling of tasks imprints on certain parameters of interests of the deployed robots (such as e.g., battery consumption). To efficiently deal with this multi-objective paradigm, a set of different multi-objective meta-heuristics, namely NSGA-II, PAES and MOHS, have been designed by using a RK encoding strategy, by which solutions simultaneously represent both the mapping from tasks to robots and the scheduling of tasks within every robot commit.

The performance of such heuristics has been assessed over three realistic scenarios deployed in Gran Canarias (Spain) in terms of different multi-objective performance indicators, which quantify the cardinality, distribution and spread of the obtained non-dominated solutions. The obtained results highlight the importance of achieving a wide Pareto front and diversity of results. In this context, both NSGA-II and MOHS attain a higher explorative behavior than PAES. Nevertheless, in terms of hypervolume MOHS renders the best performance metrics in the majority of scenarios, especially in those under operational constraints in which PAES clearly fails to explore the search space efficiently and consequently yields the worst results.

Future research will be devoted towards spanning the portfolio of algorithms in the benchmark (possibly by incorporating brand new algorithmic schemes from Swarm Intelligence and Evolutionary Computation). Furthermore, the real scenario inspiring this work calls for further constraints in the posed optimization problem, such as the inclusion of relationships of dependence between tasks, the availability of charging depots in the mission area or the transfer of unfinished tasks between robots. All these ingredients will be formulated and added to the problem statement in the near future.

## Figures and Tables

**Table 1:** Number of Non-dominant points in the resulting Pareto Front per multi-objective approach and real use case scenario.

Number of Non-Dominant Points	MOHS	NSGA-II	PAES
Baseline scenario	14	21	9
Battery-limited scenario	16	24	8
Distance-based scenario	24	7	13

**Table 2:** Normalized hypervolume (%) per multi-objective approach and real use case scenario.

Normalized Hypervolume	MOHS	NSGA-II	PAES
Baseline scenario	1.193	0.823	0.0714
Battery-limited scenario	1.132	1.075	0.0915
Distance-based scenario	0.274	0.284	0.00578

**Table 3:** Normalized hypervolume (%) with a common reference point per multi-objective approach and real use case scenario.

Normalized HV (with Common Reference Point)	MOHS	NSGA-II	PAES
Baseline scenario	1.193	0.601	1.143
Battery-limited scenario	1.132	0.697	1.174
Distance-based scenario	62.438	62.285	0.005

**Table 4:** Coverage Rate (%) per multi-objective approach and real use case scenario.

Coverage Rate (%)	MOHS	NSGA-II	PAES
Baseline scenario	0	58	31
Battery-limited scenario	0	49	34
Distance-based scenario	14	9	0
